# Dual Energy Spectral CT Imaging for Colorectal Cancer Grading: A Preliminary Study

**DOI:** 10.1371/journal.pone.0147756

**Published:** 2016-02-09

**Authors:** Hong-xia Gong, Ke-bei Zhang, Lian-Ming Wu, Brian F. Baigorri, Yan Yin, Xiao-chuan Geng, Jian-Rong Xu, Jiong Zhu

**Affiliations:** 1 Department of Radiology, Renji Hospital, Shanghai Jiao Tong University School of Medicine, Shanghai, 200127, China; 2 Department of Radiology, The University of North Carolina, Chapel Hill, North Carolina, 27516, United States of America; Northwestern University Feinberg School of Medicine, UNITED STATES

## Abstract

**Objectives:**

To assess the diagnostic value of dual energy spectral CT imaging for colorectal cancer grading using the quantitative iodine density measurements in both arterial phase (AP) and venous phase (VP).

**Methods:**

81 colorectal cancer patients were divided into two groups based on their pathological findings: a low grade group including well (n = 13) and moderately differentiated cancer (n = 24), and a high grade group including poorly differentiated (n = 42) and signet ring cell cancer (n = 2). Iodine density (ID) in the lesions was derived from the iodine-based material decomposition (MD) image and normalized to that in the psoas muscle to obtain normalized iodine density (NID). The difference in ID and NID between AP and VP was calculated.

**Results:**

The ID and NID values of the low grade cancer group were, 14.65±3.38mg/mL and 1.70±0.33 in AP, and 21.90±3.11mg/mL and 2.05± 0.32 in VP, respectively. The ID and NID values for the high grade cancer group were 20.63±3.72mg/mL and 2.95±0.72 in AP, and 26.27±3.10mg/mL and 3.51±1.12 in VP, respectively. There was significant difference for ID and NID between the low grade and high grade cancer groups in both AP and VP (all p<0.001). ROC analysis indicated that NID of 1.92 in AP provided 70.3% sensitivity and 97.7% specificity in differentiating low grade cancer from high grade cancer.

**Conclusions:**

The quantitative measurement of iodine density in AP and VP can provide useful information to differentiate low grade colorectal cancer from high grade colorectal cancer with NID in AP providing the greatest diagnostic value.

## Introduction

Colorectal cancer (CRC) has become a very common malignant tumor and one of the leading causes of cancer-related death in western countries [1] and more so in China in recent years. The 5-year survival rate following surgical intervention is approximately 50% [2]. Most studies have focused on colorectal cancer staging [3–7]. However, few studies have evaluated tumor grading. Tumor grade closely relates to degree of malignancy. A higher tumor grade suggests a poorer prognosis and reduced tumor cell differentiation. This also implies an easier ability to metastasize and higher rate of recurrence [8]. Adenocarcinoma is the most common type of colorectal cancer. According to the fourth edition of WHO Classification of Tumors of the Digestive System [9], adenocarcinoma is graded predominantly on the basis of the extent of glandular appearance, and should be divided into well, moderately and poorly differentiated types. Another method of classification is to divide the cancer into low-grade (encompassing well and moderately differentiated adenocarcinomas) and high-grade (including poorly differentiated adenocarcinomas and undifferentiated carcinomas). Poorly differentiated adenocarcinomas should show at least some gland formation or mucus production; tubules are typically irregularly folded and distorted. For this study we employed the latter classification method.

Modern imaging technology allows for detection and noninvasive staging of disease. Large tumors can be detected by conventional barium enema, while air-contrast radiography improves the visualization of less advanced lesions. Examinations such as CT, MRI, and transrectal ultrasonography, allow for the assessment of local tumor invasion and the presence of locoregional and distant metastases [10]. But the tumor grading research through CT imaging is seldom for lack of quantitative density measurement. Dual energy spectral CT (DEsCT) was recently introduced as a method of furthering diagnostic capabilities. Different from previous dual energy approaches, DEsCT employs a single x-ray tube producing dual energy spectra through rapidly alternating high and low tube voltage [11, 12]. Spectral CT produces a material decomposition (MD) image pair (e.g. water- and iodine-based material decomposition images) for accurate material density quantification. The aim of this study is to assess the feasibility and diagnostic value of dual energy spectral CT imaging for colorectal cancer grading using the quantitative iodine density measurements from MD images in both the arterial phase (AP) and venous phase (VP).

## Materials and Methods

### Patient population

This retrospective study was approved by Renji hospital institutional review board, and written informed consent was obtained. A total of 81 patients underwent CT with spectral imaging between February 2010 and October 2013 (46 men, 35 women; mean age, 62.3 years). Following CT imaging, all patients were treated surgically with standard pathologic grading. This cohort included 13 cases of well differentiated adenocarcinoma, 24 cases of moderately differentiated adenocarcinoma, 42 cases of poorly differentiated adenocarcinoma, and 2 cases of signet ring cell cancer. Undifferentiated colon adenocarcinoma patients were not included because no such cases have been collected in our study. Patients were divided into low and high grade groups based on the pathological findings. The low grade cancer group included well differentiated colon adenocarcinoma and moderately differentiated colon adenocarcinoma. The high grade cancer group included poorly differentiated colon adenocarcinoma and signet ring cell cancer.

### Patient preparation for spectral CT scan

All patients underwent standard cathartic preparation. Patients were asked to avoid products containing fiber, and to restrict their diet to fish, eggs, meat, and milk three days prior to their CT. Patients were administered polyethylene glycol oral laxatives twenty four hours before CT examination. The CT was performed following administration of a 1–1.5L gravity fed water enema (CT-WE), administered over the course of 3–4 minutes in the prone position, with distension dependent on patient comfort and tolerance.

### CT examination protocol

CT scans were performed on a Discovery CT750HD (GE Healthcare, Wisconsin, USA) scanner. The helical scan range was determined by a scout scan, and included the entire abdomen from the diaphragms to the pubic symphysis. Following the scout scan, a conventional non-enhanced helical scan was obtained at 120 kVp. Patients were then injected with a total dose of 100-140mL (1.8 mL per kilogram of body weight) of nonionic iodinated contrast material (Iopamidol 370 mg/mL; Shanghai Bracco Sine Phamaceutical Co., Ltd., China) at a rate of 3.5 mL/s followed by 50 mL saline solution using a power injector. Contrast-enhanced CT scans were performed in the arterial phase and venous phase with spectral CT imaging. Bolus tracking was utilized with the region of interest placed in the aorta, and image acquisition started 7 s after the signal attenuation reached the predefined threshold of 100 Hounsfield units (HU) for the arterial phase. A 45-second delay was utilized as the venous phase.

A pre-defined spectral CT scan protocol was used which included the following parameters: tube current 600mA, gantry rotation speed 0.6s, collimation 1.25mm, helical pitch 1.375, and scan field of view (SFOV) 50cm. Images were reconstructed in the projection space with 5mm/5mm slice thickness and interval for axial images.

### Data analysis

**Fig 1:**
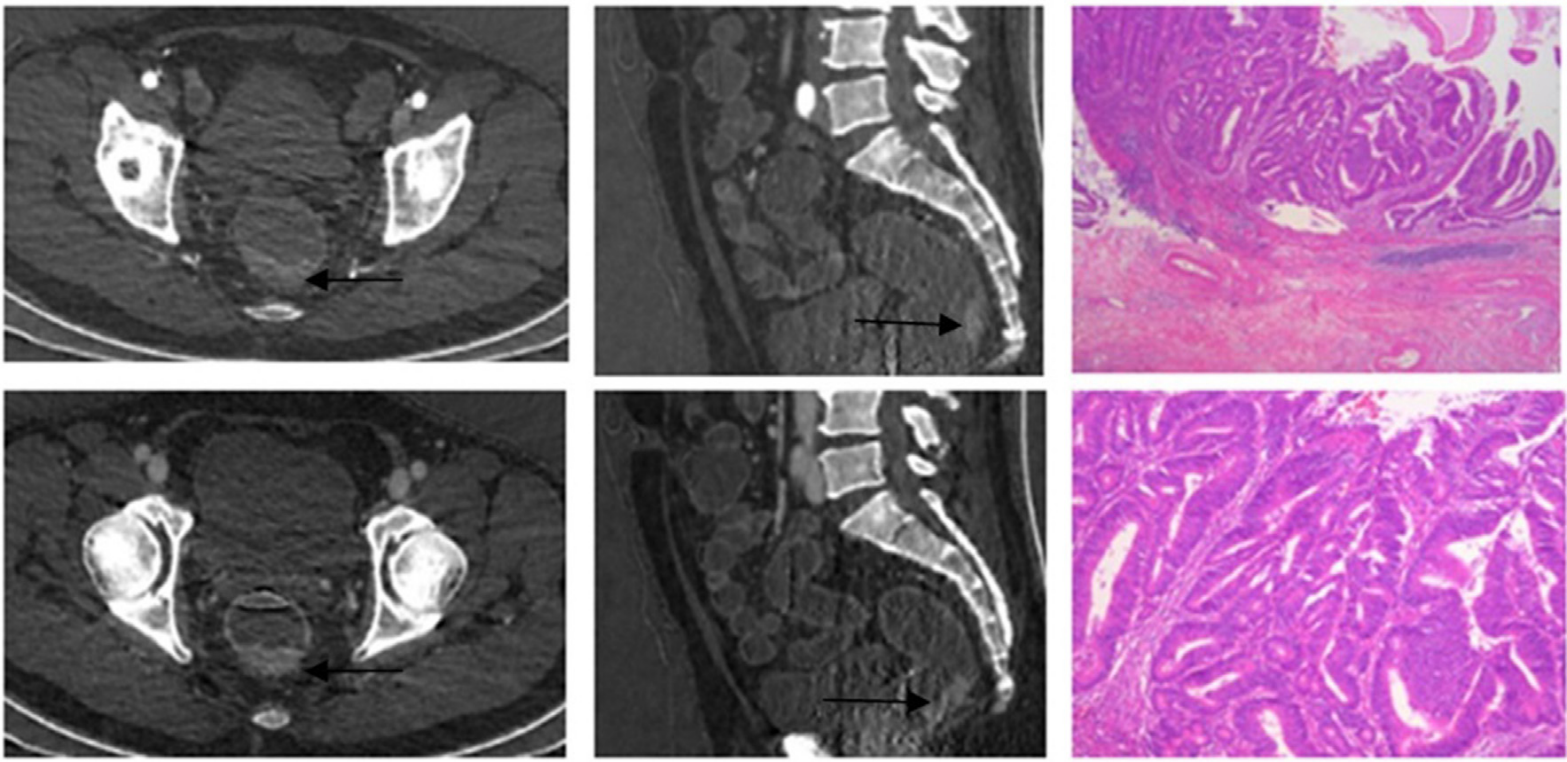
A 65-year-old man with pathologically confirmed low grade cancer(white arrow). iodine-based material decomposition images from single spectral CT acquisition (a) Coronal arterial phase, (b) Sagittal arterial phase.(c) Coronal venous phase (d) Sagittal venous phase.

**Fig 2:**
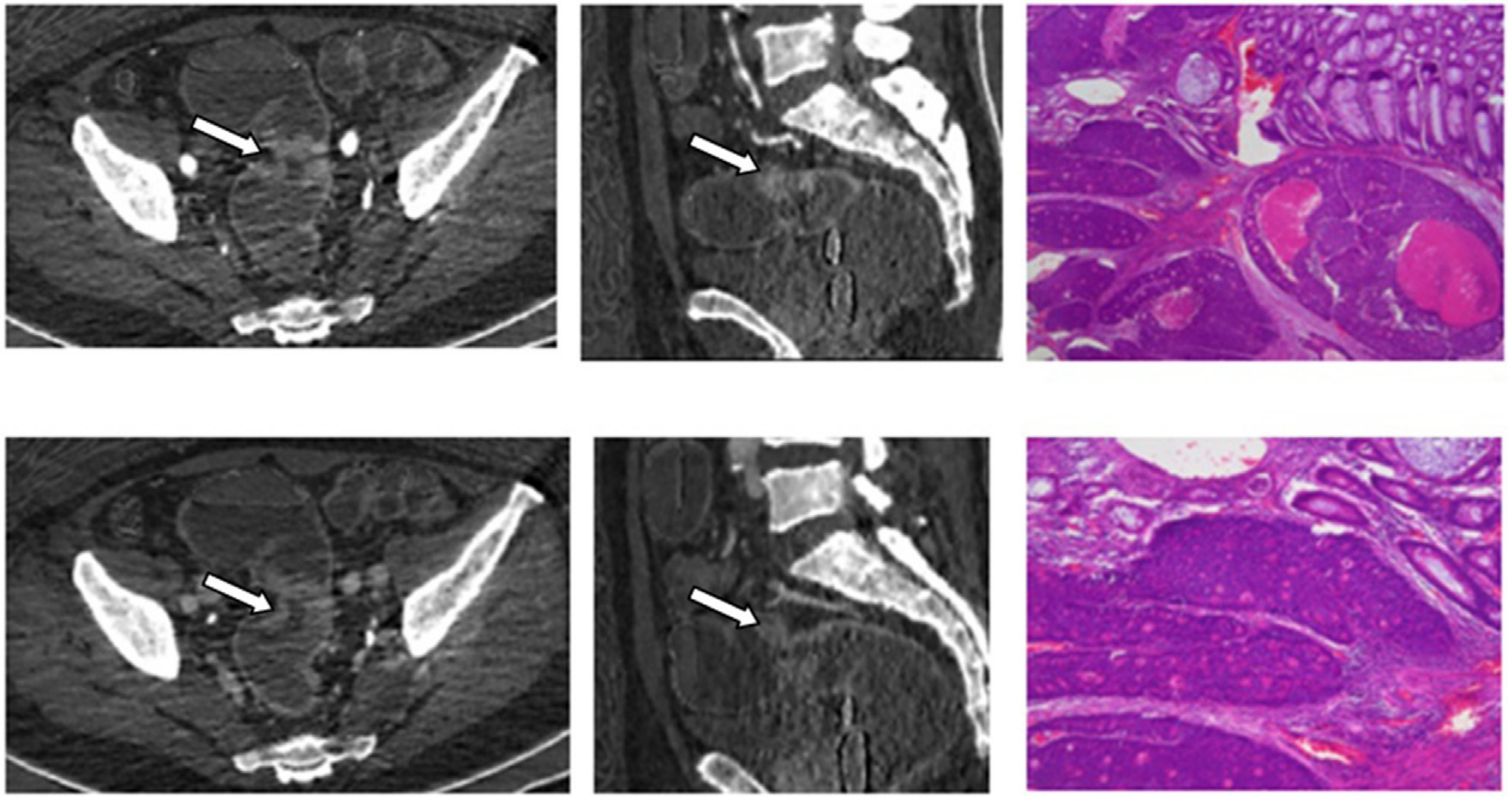
A 53-year-old female with pathologically confirmed high grade cancer (white arrow). iodine-based material decomposition images from single spectral CT acquisition (a) Coronal arterial phase, (b) Sagittal arterial phase.(c) Coronal venous phase (d) Sagittal venous phase.

All CT images were reviewed by two experienced radiologists who were blind to presenting symptoms and endoscopic results. Disagreement was infrequent and resolved by joint re-evaluation. Material decomposition images using the water- and iodine- basis material pair were reconstructed from the single spectral CT acquisition. Iodine density (ID) in the lesions was derived from the iodine-based MDCT images and normalized to the ID in the psoas muscle (normalized iodine density (NID) = ID (in lesion) / ID (in psoas)). The ROI sizes ranged from 35 mm^2^ to 55mm^2^ based on the lesion size and shape. ROIs were placed on solid regions avoiding areas with obvious features of cystic or necrotic change. Measurements were performed three times at three consecutive image levels, and mean values were calculated. Consistency in size, shape, and position of ROI between the two phases was maintained by loading the images of the double phases simultaneously into the workstation, and by using the copy and paste functions between phases. The difference of ID and NID between AP and VP in low grade cancer groups and high grade cancer groups was calculated (Figs 1 and 2).

### Statistical analysis

Iodine densities were presented as mean values ± standard deviation (SD). A two-sample t test was performed to compare the ID and NID in AP and VP of the various groups. Threshold values were created and determined by receiver operating characteristic (ROC) curves, and led to the optimal values of probabilities to differentiate patient in the low grade group from the high grade group. Diagnostic capability was determined by calculating the area under receiver operating characteristic curve. The best sensitivity and specificity, defined as the maximal sensitivity and maximal specificity values, was achieved by using the optimal thresholds. All data were analyzed using dedicated statistical software (SPSS for Windows, version 19.0). A P value of less than 0.05 was considered statistically significant.

## Results

### Colon distention

Colon distention was assessed by measuring the greatest cross-sectional diameter (from outer wall to outer wall) of each colon segment including the rectum, sigmoid colon, descending colon, transverse colon, ascending colon, and cecum. Optimal imaging signified that the segment was well distended, with uniform visualization of the intestinal wall, and a recognizable fold pattern. In our study, 97.53% (79/81) of patients were optimally distended on CT-WE.

### Quantative analysis

**Fig 3:**
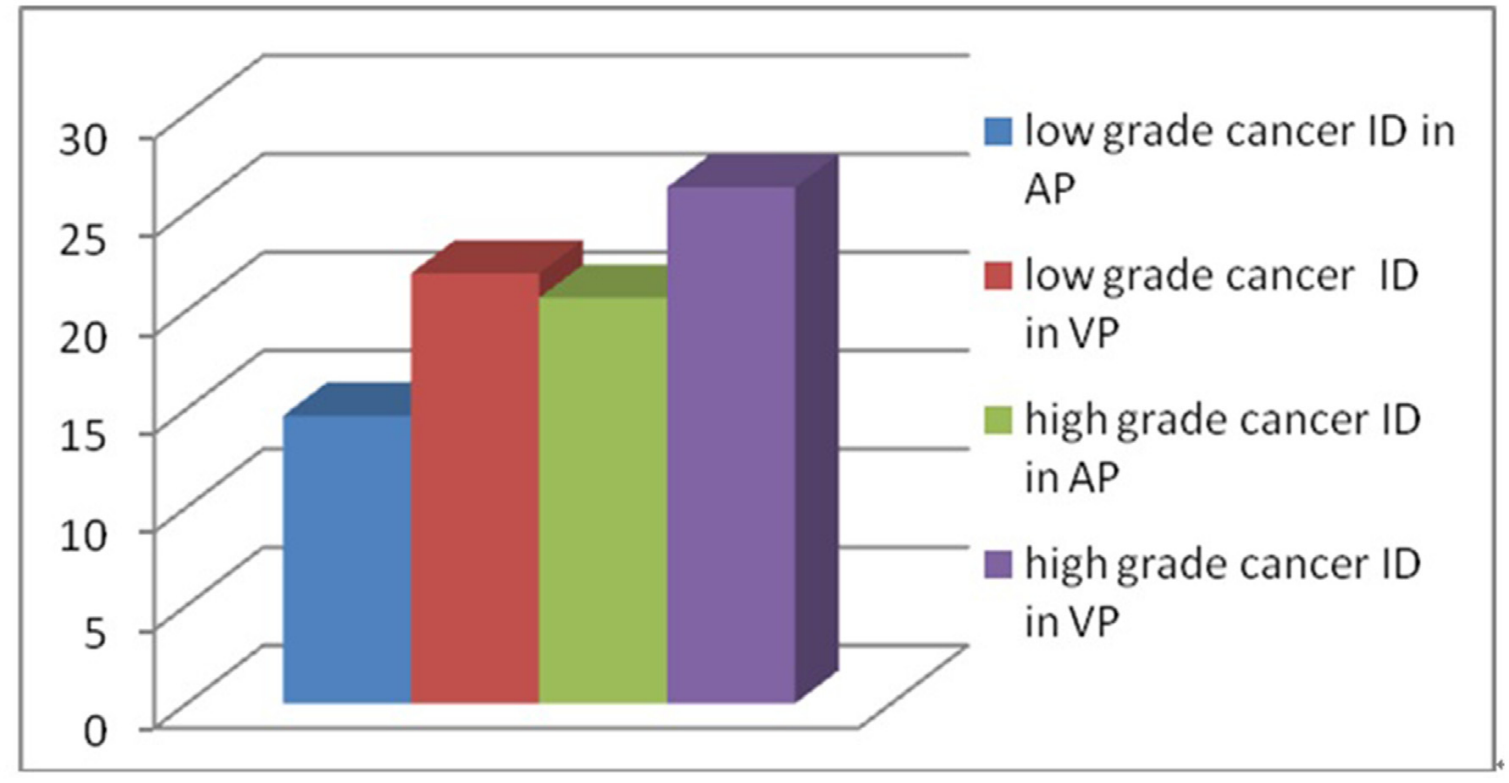
Iodine densities for the low grade cancer and high grade cancer groups.

**Fig 4:**
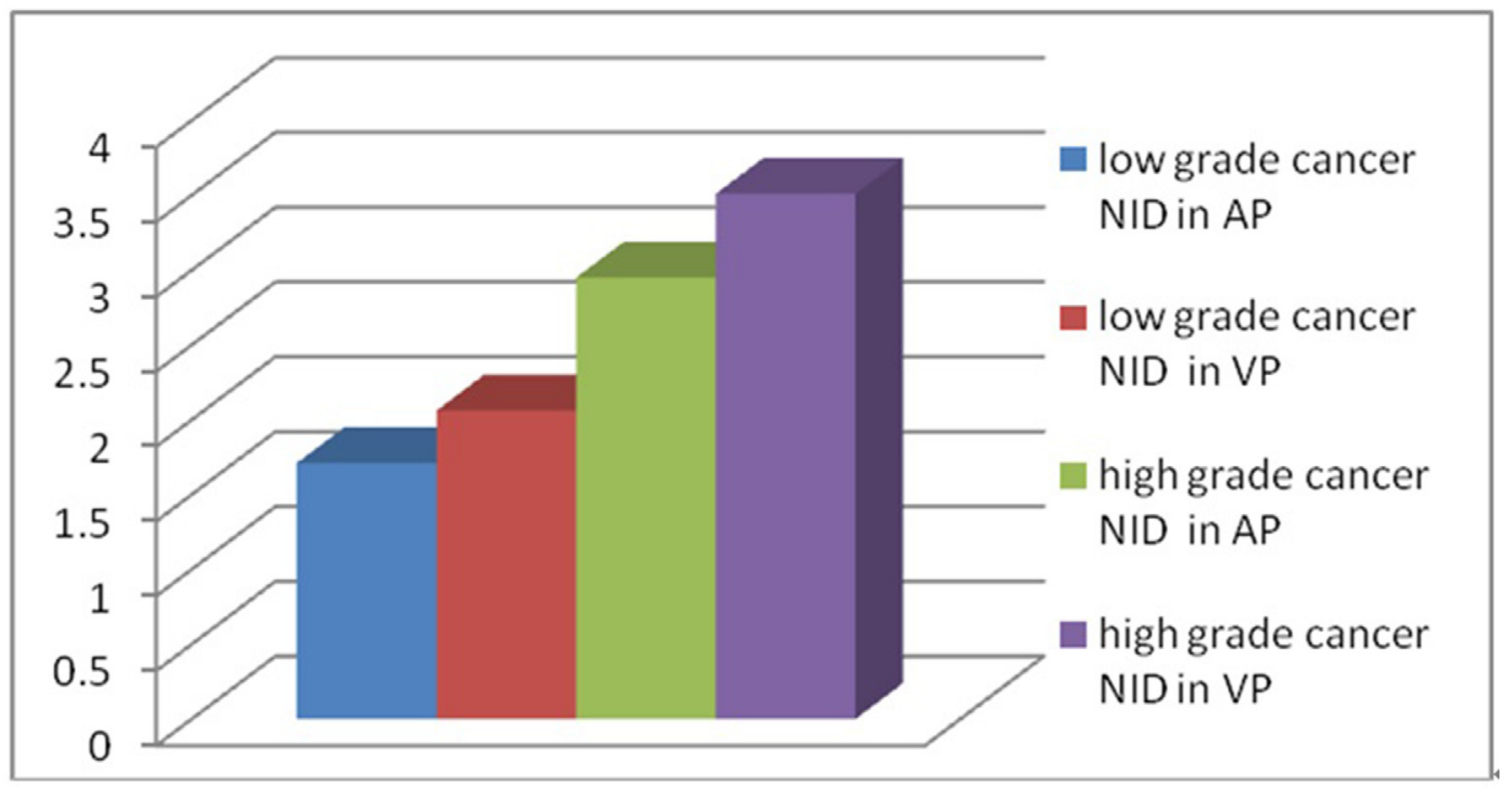
Iodine densities for the low grade cancer and high grade cancer groups.

Evaluation of low grade cancer and high grade cancer in ID and NID is shown in Figs 3 and 4. The ID and NID values of the low grade cancer were, respectively, 14.65±3.38mg/mL and 1.70±0.33 in AP, and 21.90±3.11mg/mL, 2.05± 0.32 in VP. The ID and NID values of the high grade cancer group were 20.63±3.72mg/mL and 2.95±0.72 in AP, and 26.27±3.10mg/mL and 3.51±1.12 in VP, respectively. There was a significant difference for ID and NID between the low grade and high grade cancer groups in both AP and VP (all p<0.001).

**Fig 5:**
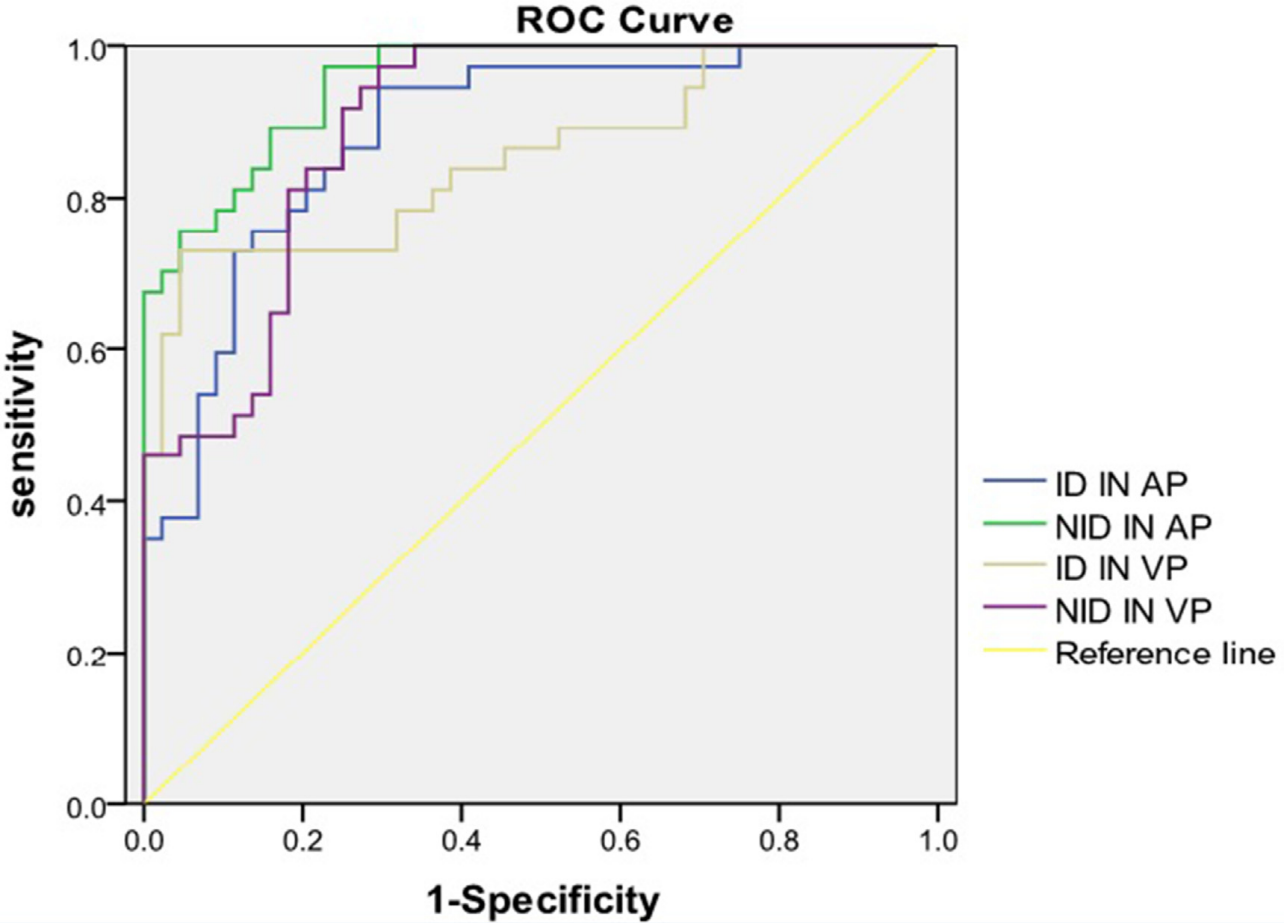
Receiver operating characteristic (ROC) curve of colon cancer grading. ROC analysis indicated that normalized ID (NID) of 1.92 in arterial phase (AP) provided 70.3% in sensitivity and 97.7% in specificity in differentiating low graded cancer from high grade cancer with the area-under-curve (AUC) of 0.953.

Using the ROC analysis, we obtained the thresholds for ID and NID to optimize both the sensitivity and specificity for differentiating low grade cancer from high grade cancer. We compared ROC curves of ID and NID both in AP and VP (Fig 5, Table 1). Among them, the NID in AP provided the highest value of the area under the curve (AUC) for ROC study (p<0.001). Using the threshold value of 1.92 for NID in AP, one could obtain the sensitivity of 70.3% and the specificity of 97.7% with AUC = 0.95.

**Table 1:** The area under the curve (AUC) for ROC study.

Item	AUC	P	95% confidence interval
ID in AP	0.886	0.000	0.814–0.957
ID in VP	0.853	0.000	0.767–0.939
NID in AP	0.953	0.000	0.914–0.991
NID in VP	0.895	0.000	0.828–0.961

## Discussion

Colon cancer is the most common digestive cancer in many countries [13–17]. Diagnosis is usually based on invasive colonoscopy, which provides direct visualization of lesions and allows for biopsies. Recent improvements in CT have allowed for minimally invasive large bowel evaluation at a substantial reduction in cost and patient risk. A critical technical requirement for CT evaluation of large bowel is full distension of a cleansed lumen with complete separation of the intestinal walls. In our study all patients received CT-WE for bowel distension, with 97.53% optimally imaged. Two patients failed to undergo optimal imaging due to an unsuccessful enema. Because these two patients’ colon lesions were obvious, diagnosis was not affected. Luminal collapse or incomplete colonic filling may lead to false-negative outcomes as polyps and small colorectal tumors can be obscured [18]. However, CT-WE offered excellect visualization of the colonic wall due to parietal enhancement by iodine contrast, as well as good contrast between wall, water-filled lumen, and pericolic fat [19].

Unlike conventional CT imaging which produces only polychromatic CT-number images, dual energy spectral CT acquisition is a novel imaging technique which generates monochromatic and material decomposition images. This is accomplished by quickly alternating high and low tube voltages on adjacent views during gantry rotation. The monochromatic images provide better contrast resolution than conventional polychromatic images, whereas material decomposition images offer material density measurement. Quantitative iodine density measurement may be used to differentiate low and high grade colorectal carcinoma. Although scanning parameters were consistent in all patients, individual differences were present. In order to minimize these differences, NID was calculated except absolute iodine density. According to our results, low grade cancer’s ID in AP and VP were 14.65±3.38mg/mL and 21.90±3.11mg/mL, respectively. Low grade cancer’s NID in AP and VP were 1.70±0.33 and 2.05± 0.32, respectively. High grade cancer’s ID in AP and VP were 20.63±3.72 mg/mL and 26.27±3.10 mg/mL, respectively. High grade cancer’s NID in AP and VP were 2.95±0.72 and 3.51± 1.12, respectively. There was significant difference for ID or NID between low grade and high grade cancer in AP or in VP (p<0.001). High grade cancer has higher iodine density than low grade cancer. Other parts of the tumor had the similar reports about blood supply and tumor grading [20, 21]. Using the ROC analysis, we obtained the thresholds for ID and NID to optimize both the sensitivity and specificity for differentiating low grade from high grade cancer. Receiver operating characteristic analysis revealed that the area under the ROC curve for NID in AP was greatest. The diagnostic value of NID in AP was best for differentiating low grade and high grade cancer. When diagnostic threshold of NID in AP was 1.92, the sensitivity was 70.3% and the specificity was 97.7%. The diagnostic value of NID in AP was superior to the other three values for differentiating low grade from high grade cancer.

The majority of previous radiological studies evaluating tumor grading involves MRI imaging [22–24]. Pathological angiogenesis, triggered by activation of certain cellular signal pathways, is considered a key factor for solid tumors to develop, grow, and metastasize [25]. Colorectal carcinoma has been found highly associated with angiogenesis, and malignant rectal tumor tissue generally has greater angiogenic activity than normal rectal tissue [26–29]. GSI is a noninvasive imaging technique demonstrating the iodine density variance between low grade and high grade cancer groups. Iodine density was associated with contrast concentration in vessels which may reflect blood vessel density, tumor activity, and invasion. As such, GSI is a potential tool for exposing angiogenesis and metabolic activity in rectal adenocarcinoma. Material decomposition images in spectral CT imaging generate quantitative material density, such as iodine density. These measurements may provide an alternative method to directly evaluate tumor treatment and chemotherapy response.

Several limitations exist in this study. First, this investigation reflects our preliminary results in a small sample of patients. In the low grade group, only poorly differentiated colon adenocarcinoma and signet ring cell carcinoma were included. Other tumors such as medullary carcinoma, carcinosarcoma, etc, were not considered. This may influence results. Second, further clinical trials need to be performed to confirm our quantitative data since iodine density may be influenced by injection parameters and cardiopulmonary status. This was in part offset by using the normalized iodine density which minimized individual differences and thereby reduced error. Third, the ROIs were placed as far intralesionally as possible to improve accuracy, however slice selection may influence outcomes. Although the average iodine density value was calculated, measurement deviation cannot be absolutely avoided. Last, this study was focused on the use of quantitative measurement generated by using CT spectral imaging. The quantitative assessment of iodine- and water-based images could be assessed in future studies to determine their clinical value.

## Conclusions

Spectral CT imaging generated material decomposition images for quantitative depiction of colorectal carcinoma. The quantitative measurement of iodine density and normalized iodine density in AP and VP can provide useful information to differentiate low grade tumors from high grade tumors. NID in AP was superior to the other three values, and provided more information for clinical diagnosis and consequently treatment.
